# PIM1 induces hypoxia-related fibroblast senescence in a mouse model of stress urinary incontinence

**DOI:** 10.1371/journal.pone.0335501

**Published:** 2025-11-12

**Authors:** Ya Xiao, Mao Chen, Lingyun Li, Liying Chen, Xiaoyu Tian, Xiaoyu Huang, Fangyi Zhu, Bingshu Li, Li Hong

**Affiliations:** 1 Department of Gynecology and Obstetrics, Renmin Hospital of Wuhan University, Wuhan, Hubei, China; 2 Guangdong Provincial People’s Hospital, Guangzhou, China; 3 Pelvic Floor Research Centre of Hubei Province, Renmin Hospital of Wuhan University, Wuhan, China; Colorado State University, UNITED STATES OF AMERICA

## Abstract

**Objective:**

This study aims to explore the contribution of PIM1 kinase-mediated cellular senescence to the pathogenesis of stress urinary incontinence (SUI) and to assess the therapeutic potential of inhibiting PIM1.

**Methods:**

A mouse model of SUI was developed through vaginal balloon dilation to investigate hypoxia in the vaginal wall, utilizing HypoxyprobeTM-1 staining and HIF-1α expression analysis. Cellular senescence was evaluated by measuring SA-β-gal activity, senescence-associated heterochromatin foci (SAHF) formation, Ki67 expression, and γH2A.X accumulation. In vitro experiments involved the use of hypoxia-treated fibroblasts subjected to PIM1 knockdown or treatment with AZD-1208. Functional outcomes were assessed through bladder leak point pressure tests and histological analysis.

**Results:**

The study revealed that the SUI model exhibited significant reductions in vaginal wall blood flow and an increase in hypoxia markers. Indicators of cellular senescence were significantly elevated in SUI tissues, alongside a notable upregulation of PIM1. Mechanistically, PIM1 facilitated senescence through two pathways: inducing cell cycle arrest via activation of P16/P21 and impairing DNA repair through the formation of SAHF. Hypoxic conditions significantly enhanced PIM1 expression and senescence markers in fibroblasts, effects that were effectively reversed by PIM1 inhibition. Treatment with AZD-1208 led to significant improvements in bladder function and a reduction in senescence burden in vivo.

**Conclusion:**

This study identifies PIM1 as a critical mediator linking hypoxia-induced cellular senescence to the development of SUI. The PIM1 inhibitor AZD-1208 demonstrates promising therapeutic effects, offering a novel approach for the treatment of SUI, with particular relevance for postpartum prevention. These findings elucidate a comprehensive hypoxia-PIM1-senescence pathogenic pathway and identify a potential target for clinical intervention.

## 1. Introduction

Stress urinary incontinence (SUI) is a significant disorder affecting the female population, characterized by an increase in intra-abdominal pressure due to various factors such as exertion, coughing, or sneezing, which triggers involuntary urine leakage [1]. Recent epidemiological evidence suggests that the prevalence of SUI ranges between 10% and 39% across studies, with an increase observed with advancing age [2–4]. Data released by the U.S. Census Bureau indicates that the demand for pelvic floor disorder care is projected to rise by 35% between 2010 and 2030 [5]. Additionally, SUI not only adversely impacts women’s quality of life but also results in a loss of productivity, which, in turn, negatively affects families and socioeconomics [6]. Thus, SUI has a profound impact on women’s health and quality of life, leading to significant physical and mental stress and incurring substantial costs within the healthcare system and women’s personal finances. However, the pathogenesis of SUI remains incompletely understood. Therefore, further investigation into the pathogenesis of SUI is essential for the advancement of disease prevention and treatment modalities.

The etiology of SUI in women is multifactorial, with trauma from childbirth identified as a significant risk factor [7]. Specifically, the risk of SUI nearly doubles following vaginal delivery compared to cesarean delivery [8]. In a study involving over 15,000 women, 12.2% of those who delivered vaginally developed SUI, in contrast to 4.7% of women who did not give birth and 6.9% of those who underwent cesarean section [9]. The trauma associated with vaginal delivery not only damages pelvic floor structures but also results in nerve injury to the vagina and urethra. Key risk factors linked to these adverse outcomes include prolonged duration of the second stage of labor, high fetal birth weight, and multiple pregnancies. Vaginal dilation during labor can lead to reduced blood flow and hypoxia affecting the bladder, urethra, and vagina. Research indicates that ischemia-related tissue hypoxia due to stress during the second stage of labor contributes to the pathogenesis of SUI [10]. Additionally, age has been recognized as a significant risk factor for SUI; menopause or advanced age may lead to physiological senescence of periurethral tissues, while hypoxic injury can induce premature senescence of urethral support tissues. This provides a systematic explanation for the prevalence of SUI in elderly individuals, particularly those experiencing high abdominal pressure factors such as pregnancy, labor and delivery, coughing, and constipation, as well as in premenopausal patients. Further investigation is warranted to determine whether cellular senescence associated with vaginal delivery contributes to the development of SUI.

Cellular senescence is a state characterized by a permanent arrest of the cell cycle and a range of phenotypic changes, triggered by various stressors, including genotoxic agents, nutrient deficiencies, hypoxia, mitochondrial dysfunction, and oncogene activation [11,12]. Research has demonstrated that cellular senescence serves to prevent or inhibit the proliferation of damaged or dysfunctional cells, thereby playing a protective role in disease development. However, the aberrant accumulation of senescent cells in tissues can lead to detrimental effects, such as the overproduction of senescence-associated secretory phenotype (SASP) factors through a paracrine mechanism. The SASP can induce senescence in neighboring healthy cells, creating a feedback loop that exacerbates tissue dysfunction and accelerates the aging process [11]. Senescent cells exhibit various molecular features and cytological markers, including enlarged and flattened morphology, elevated senescence-associated β-galactosidase activity (SA-β-gal), activation of the p53 and p21Cip1/WAF1 pathways, a senescence-associated secretory phenotype, and the presence of senescence-associated heterochromatin foci (SAHF) [13–15].SAHF are formed through the condensation of chromatin into distinct punctate regions, which in turn repress the expression of genes encoding proteins associated with cell cycle progression, such as cytosolic protein A, proliferating cell nuclear antigen, and cytosolic protein D1. Recent studies have indicated that senescent fibroblasts accumulate in pelvic tissues, gradually depleting these tissues of proliferative and regenerative stem cells, thereby disrupting their homeostatic and regenerative capacities. As the number of active fibroblasts declines, pelvic tissue loses the collagen necessary to support the pelvic organs [16]. However, to date, there has been limited exploration of the effects of cellular senescence on SUI, and the underlying molecular mechanisms remain largely unknown.

PIM 1 kinase is a constitutively active serine/threonine kinase that plays a critical role in regulating various cellular processes, including proliferation, apoptosis, differentiation, and metabolism. PIM1 only contributes to cell proliferation and survival but also influences senescence by modulating the cell cycle and apoptosis [17]. For instance, PIM 1 kinase promotes cellular senescence through the phosphorylation of the high mobility group protein box transcription factor 1 (HBP1), a mechanism that is associated with the cellular response to oxidative stress [18]. Furthermore, phosphorylation of CBX8 by PIM1 can lead to its destabilization and degradation, thereby promoting P16 expression during oncogene-induced cellular senescence [19].

Consequently, we are interested in investigating whether PIM1 is implicated in the regulation of SUI pathogenesis.

This study successfully developed an animal model of vaginal delivery to systematically elucidate, for the first time, the critical role of PIM1 in the pathogenesis of SUI. Our findings indicate that a hypoxic microenvironment upregulates PIM1 expression, which subsequently facilitates the formation of SAHF and activates the P16/P21 pathway. This process ultimately leads to the senescence and functional decline of pelvic floor supportive tissues. Notably, we identified that the PIM1 inhibitor AZD-1208 effectively reverses this pathological process, thereby offering a novel targeted therapeutic strategy for SUI. These findings not only enhance our understanding of SUI pathogenesis but also provide a theoretical foundation for the development of innovative treatment approaches focused on senescence intervention.

## 2. Methods

### 2.1. Source of data

The gene expression profile of GSE53868 (GEO Accession viewer) was obtained from the Gene Expression Omnibus (GEO) database [20], a public repository hosting numerous high‐throughput sequencing and microarray data sets contributed by research institutions globally. A total of 866 genes were previously confirmed to be associated with senescence and were obtained from the GeneCards database [21]. A total of 4178 chromatin regulators and 799 heterochromatin regulation were retrieved from previous research [22].

### 2.2. Bioinformatics analyses

Differential expression analysis was performed using the DESeq2 package in the R program, and was visualized using the ggplot2 package with a threshold of p < 0.05 and LogFC > 1.5. Venn diagrams were generated by Draw Venn Diagram (https://bioinformatics.psb.ugent.be/webtools/Venn/). To gain a deeper understanding of the biological processes linked to DFU, we conducted Gene Set Enrichment Analysis (GSEA) using the clusterProfiler package [23]. Hallmark and Canonical Pathways gene sets were obtained from the MSigDB collections (https://www.gsea‐msigdb.org/gsea/msigdb/index.jsp). Adjusted *p*‐value <0.05 and FDR (qvalue) < 0.25 were regarded as the cut‐off criteria. To gain a deeper understanding of the primary biological functions of CS‐DEGs, we employed the clusterProfiler package for analysing the Gene Ontology (GO) and Kyoto Encyclopedia of Genes and Genomes (KEGG) pathways that regulate CS‐DEGs both up and down. An adjusted p‐value <0.05 was deemed significant.

### 2.3. SUI mouse model

Eight-week-old female C57BL/6 mice were obtained from the Laboratory Animal Center of Wuhan University and maintained under standard conditions (20 ± 2°C, 40–50% humidity, 12-h light/dark cycle) with free access to food and water, in accordance with the institutional guidelines for animal care and use. The mice were randomly divided into three groups (n = 6/group): sham-operated controls (CON), vaginal distension for 1 h (VD 1h), and vaginal distension for 4 h (VD 4h). Three days prior to modeling, selected mice received daily intraperitoneal injections of AZD1208 (40 mg/kg, HY-15604, MedChemExpress) or vehicle control (10% DMSO in 90% saline) and continued throughout the study period [24]. For surgical procedures, mice were anesthetized with 3% isoflurane (maintained at 1.5%) and placed on a heating pad, followed by aseptic preparation and insertion of a modified 6-Fr Foley catheter (tip removed and integrity verified) after lubrication; the catheter was secured with 5/0 silk sutures, with experimental groups receiving 0.3 mL saline inflation (8 mm diameter) for either 1 h or 4 h, while sham controls underwent catheter placement without inflation for 1 h. Postoperatively, mice were individually housed and administered buprenorphine (0.1 mg/kg, subcutaneously) for analgesia, with daily monitoring of surgical sites. At study endpoint, euthanasia was performed via graded CO2 asphyxiation (30% chamber replacement rate initially, increased to 100%) followed by cervical dislocation, with model efficacy assessed through urodynamic measurements including leak point pressure and bladder capacity; all procedures were conducted independently by two experienced investigators to ensure consistency and reproducibility.

### 2.4. Mouse urodynamic testing

The mice were anesthetized using isoflurane and subsequently positioned in a supine orientation. The pediatric indwelling needle was removed from the core, saline was injected to facilitate urine ventilation, and the surface was lubricated with bupivacaine paste prior to catheterization. The catheterization technique involved one hand holding micro forceps to clamp the external urethral orifice and elevate it to straighten the urethra, while the other hand gently inserted the indwelling needle into the urethra to a depth of approximately 1 to 1.2 cm. Following successful catheterization, the empty needle was utilized for suction to empty the bladder of the mice, and extension tubing along with a tee was attached, connecting to a manometer tube and a microsyringe pump. Once the tubing was successfully connected, the body was zeroed, and the microinjection pump was activated to instill saline into the bladder at a rate of 1 mL/h. Bladder pressure changes were monitored, and the mice were observed for urine overflow; at this point, the recorded pressure was designated as bladder leak point pressure (BLPP). The BLPP was documented upon the appearance of the first drop of liquid at the urethra, and the entire experimental procedure was repeated three times. After completion of the tests, the external urethral opening was sterilized using iodophor.

### 2.5. High-resolution laser speckle contrast imaging system(HR-LSCI)

**Fig 1:**
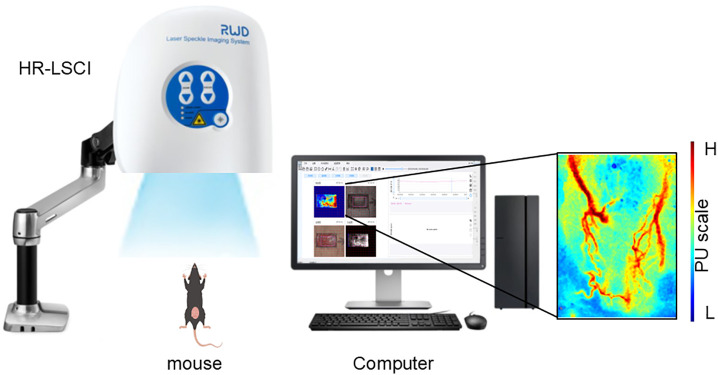
Laser speckle contrast imaging modality setup. (A)Schematic of imaging modality. HR-LSCI: high resolution laser speckle contrast imaging; (B) A LSCI of vaginal wall blood flow in a female mouse. PU: perfusion units, PU scale: red = high flow, blue = low flow.

In this study, the HR-LSCI Laser Scattered Blood Flow Imaging System (RWD Life Technologies, Shenzhen, China) was utilized, as illustrated in Fig 1, which presents a schematic diagram of the imaging modalities of the HR-LSCI system. A 780 nm dispersive laser beam was employed to illuminate the region of interest in the vaginal wall, resulting in the formation of a speckle pattern within the irradiated area. Blood flow changes were analyzed using the middle section of the vaginal wall as the region of interest (ROI). The scatter pattern of the illuminated area was monitored with an sCMOS camera, which had a resolution of 1472 × 1104 pixels, and maintained consistent parameters across all photographs (display rate: 25 Hz; time constant: 1 s; exposure time: 10 ms; intensity: 60 mA; resolution: 2048 × 2048). Furthermore, the imaging speed of the LSCI system exceeded 100 fps, with an acquisition detection rate of two images per second. The signals acquired were transformed into two-dimensional blood perfusion maps through the HR-LSCI algorithm, which converted the blood perfusion information into these maps. Image processing and analysis were conducted using the system’s integrated software, which presented the recorded values in perfusion units (PU).

### 2.6. Hypoxyloprobe labeling

Hypoxyprobe staining (Hypoxyprobe™ RedAPC, Natural Pharmacia International) was conducted on day 7 following vaginal delivery (VD) modeling to assess hypoxic damage to the vaginal wall in mice post-VD. Hypoxyprobe-1, a 2-nitroimidazole (pimonidazole hydrochloride) derivative, is water-soluble and widely utilized to illustrate hypoxic conditions in both in vivo tissues and cultured cells (2, 10). Upon injection into experimental animals or humans, Hypoxyprobe-1 is rapidly distributed throughout all tissues in vivo; however, it forms adducts with proteins exclusively in cells where oxygen concentrations fall below 14 μM (equivalent to 10 Torr of Po2 at 37°C) [10]. Prior to perfusion, the animals received an intraperitoneal injection of Hypoxyprobe-1 solution (pimonidazole hydrochloride dissolved in 0.9% NaCl) at a dosage of 60 mg/kg. Sixty minutes post-injection, tissues from the vaginal wall of the mice were fixed in 4% paraformaldehyde, embedded in paraffin, and subsequently sectioned for immunohistochemistry. The probe antibody was directly labeled with APC, allowing for direct use in immunofluorescence detection. For cell samples, 200 μM Hypoxyprobe-1 was incubated with the cells for 1 hour and then fixed in 4% paraformaldehyde for subsequent immunization. DAPI was employed to detect cell nuclei.

### 2.7. Beta-galactosidase staining

Senescence-associated β-galactosidase activity assay was performed according to the manufacturer’s instructions (C0602, Beyotime) to measure cellular senescence. Observation and photography were performed with a microscope.

### 2.8. Immunofluorescence

In this study, immunofluorescence techniques were utilized to investigate both tissue and cellular samples. For tissue immunofluorescence, sections of paraffin-embedded mouse vaginal wall tissue, each 4 μm thick, were prepared. These sections were blocked with 5% horse serum at room temperature for 60 minutes, followed by overnight incubation at 4°C with primary antibodies targeting HP1γ(1:200 dilution, A2248, Abclonal), H3K9me3 (1:200, A2360, Abclonal) [25], and γH2A.X (1:100, WL00616a, Wanleibio) diluted in PBS. After washing with PBST, the sections were incubated with CY3/FITC-conjugated secondary antibodies for 1 hour at room temperature, counterstained with DAPI (1:500, D8200, Solarbio) for nuclear visualization, mounted, and imaged randomly using an Olympus BX53 fluorescence microscope. For cellular immunofluorescence, L929 cells cultured on coverslips were fixed with 4% paraformaldehyde for 15 minutes, permeabilized with 0.3% Triton X-100, and blocked with 5% normal goat serum for 15 minutes, followed by a 1-hour blocking period. Subsequently, the cells were incubated overnight at 4°C with primary antibodies against HP1γ (1:200), H3K9me3 (1:200), and PIM1 (1:200, A19695, Abclonal). The secondary antibody incubation and staining procedures were conducted in a manner identical to those used for tissue samples.

### 2.9. Immunohistochemistry

In this study, standard immunohistochemical analysis was conducted on mouse vaginal wall tissues. Paraffin-embedded tissue samples were sectioned into 20-μm -thick cross-sections and incubated with primary antibodies targeting PIM1 (dilution 1:200, Cat# A19695, Abclonal) and Ki67 (dilution 1:100, Cat# A20018, Abclonal) at room temperature for a duration of 2 hours. This was followed by a 1-hour incubation with species-matched secondary antibodies. Upon completion of the staining process, the sections were mounted using ProLong Antifade Mountant (Invitrogen). Quantitative analysis was performed by measuring optical density, calculated as (intensity of the region of interest – mean intensity of the whole image)/ mean intensity of the whole image × 100%, with the results expressed as percentages.

### 2.10. Cell culture and processing

In this study, L929 mouse fibroblast cells, obtained from Procell, were cultured in minimum essential medium (MEM, Procell) supplemented with 10% fetal bovine serum under standard conditions (37°C, 5% CO2). Cells were passaged or cryopreserved upon reaching approximately 80% confluence. To establish chemical hypoxia models, the cells were treated with 200 μM cobalt chloride (CoCl_2_, Huishi, 10007216 AR) for 12 or 24 hours. Physical hypoxia models were generated using a hypoxia workstation set to 1% O2 concentration, with experimental groups designated as normoxic control, 24-hour hypoxia, and 48-hour hypoxia [26]. For studies on PIM1 kinase inhibition, the specific inhibitor AZD1208 (HY-15604, Med Chem Express) was dissolved in dimethyl sulfoxide (DMSO) and administered at concentrations of 10 μM or 20 μM, with a vehicle control containing 0.1% DMSO. All treated samples were collected following appropriate incubation periods in accordance with subsequent experimental requirements.

### 2.11. Western blotting

Protein samples derived from tissues and cells were lysed in pre-chilled RIPA buffer, which was supplemented with protease and phosphatase inhibitors for a duration of 10 minutes. This was followed by centrifugation at 12,000 rpm for 10 minutes at 4°C. Protein concentrations were quantified using a BCA protein assay kit (P0006, Beyotime Biotechnology, China). Post-denaturation, the protein samples were subjected to separation via SDS-PAGE and subsequently transferred onto PVDF membranes. These membranes were blocked with 5% skim milk in TBST for 1 hour and incubated overnight at 4°C with primary antibodies at the following dilutions: PIM1 (1:1000, Abclonal, #A19695), P21 (1:1000, Wanleibio, #WL0362), P16 (1:3000, ELK Biotechnology, #ES8349), LaminB1 (1:1000, Abclonal, #A1910), γH2A.X (1:1000, Wanleibio, #WL00616a), HIF-1α (1:1500, Abclonal, #A26889), and β-actin (1:20000, Proteintech, #66009–1-Ig). Subsequently, the membranes were incubated for 1 hour at room temperature with an HRP-conjugated affinity-purified goat anti-rabbit IgG (H + L) secondary antibody (1:10000, Proteintech, #SA00001–2). Protein bands were visualized using the enhanced chemiluminescence (ECL) method on a Bio-Rad imaging system, and band intensities were quantified using ImageJ software.

### 2.12. RT-qPCR

Tissue and cell samples were extracted for total RNA using the RNA Extraction Kit (R0027, Beyotime). A total of 1000 ng of RNA was reverse transcribed into cDNA with the cDNA Synthesis Kit (G3330-100, Servicebio). Real-time quantitative PCR (RT-qPCR) was conducted on a Bio-Rad CFX Connect using SYBR Green PCR Master Mix (G3326-01, Servicebio). The RT-qPCR primers utilized in this study are detailed in Supplementary Table 1 (S1 Table).

### 2.13. Overexpression of PIM1

The PIM1 overexpression plasmid was synthesized and purchased from MIAOLING BIOLOGY (Wuhan, China). L929 cells cultured in MEM were transfected with either pPIM1 or pVector using Optimem medium in conjunction with Lipofectamine 2000 (#11668019, Invitrogen). Six hours post-transfection, the Optimem medium was replaced with fresh MEM medium supplemented with 10% FBS. The effectiveness of overexpression was subsequently assessed using RT-qPCR andwestern blot.

### 2.14. Transfection of siRNA

The transfection of siRNA was conducted by GENERAL BIOL (Anhui, China), which designed and synthesized PIM1-siRNA (si-PIM1) and negative control siRNA (NC-siRNA, si-NC). The target sequences of the siRNAs were as follows: si-PIM1, “CAAGUGUUCUUCAGGCAAATT”; si-NC, “UUCUCCGAACGUGUCACGUTT”. L929 cells, cultured in MEM supplemented with 10% FBS, were transfected with either si-PIM1 or si-NC using Optimem medium and Lipofectamine 2000 (#11668019, Invitrogen). After 6 hours, the Optimem medium was replaced with fresh MEM medium containing 10% FBS, and the effect of the RNA intervention was assessed using RT-qPCR.

### 2.15. Wound healing assay

Cells were seeded in 6-well plates and cultured until reaching approximately 90% confluence. A sterile 200 μL pipette tip was used to create a uniform scratch in the cell monolayer. After gently washing twice with PBS to remove detached cells, the cells were subjected to respective treatments, including CoCl₂-induced chemical hypoxia or physical hypoxia in a tri-gas incubator at 1% O₂. Images of the same scratch region were captured at 0, 24, and 48 hours using an Olympus IX51 inverted microscope (Olympus, Japan) at 100 × magnification. The scratch area at each time point was delineated and quantified using Image J software (National Institutes of Health, USA). Cell migration ability was evaluated by comparing the changes in scratch area over time.

### 2.16. Transwell migration assay

Cell migration was assessed using Transwell chambers (Corning, USA, Cat. No. 3422). Briefly, cells were trypsinized, counted, and resuspended in serum-free medium containing the corresponding treatments (CoCl₂ or AZD-1208). Then, 5 × 10⁴ cells per well were seeded into the upper chamber. The lower chamber was filled with 600 μL of complete medium containing 10% FBS as a chemoattractant. After 24 hours of incubation at 37°C with 5% CO₂, non-migrated cells on the upper surface of the membrane were carefully removed with a moistened cotton swab. Migrated cells on the lower surface were fixed with 4% paraformaldehyde and stained with 0.1% crystal violet. The membranes were visualized under an Olympus IX71 inverted microscope (Olympus, Japan) at 100 × magnification. Cells in three randomly selected fields were counted using Image J software.

### 2.17. Cell cycle analysis

Cell cycle distribution was analyzed by propidium iodide (PI) staining and flow cytometry. A commercial Cell Cycle and Apoptosis Analysis Kit (Beyotime, China, Cat. No. C1052) was used according to the manufacturer’s instructions. Cells were seeded in 6-well plates at a density of 2 × 10⁶ cells per well and treated as indicated. After treatment, cells were harvested, washed with ice-cold PBS, and fixed overnight in 75% ethanol at 4°C. Fixed cells were washed again, resuspended in 500 μL of PI/RNase staining buffer, and incubated in the dark at room temperature for 30 minutes. DNA content was analyzed using a CytoFLEX flow cytometer (Beckman Coulter, USA), and cell cycle distribution was determined with FlowJo software (TreeStar, USA).

### 2.18. Statistical analyses

Statistical analyses and data visualization were conducted using Prism software (version 8.0). All data are expressed as the mean ± standard deviation. To assess significance, Student’s t-test was applied for two groups, while one-way analysis of variance (ANOVA) followed by Bonferroni’s test was utilized for multiple groups. All statistical tests were two-tailed, with a p-value of less than 0.05 considered significant.

## 3. Results

### 3.1. Simulated delivery induces hypoxic injury and cellular senescence in the vaginal wall of mice

**Fig 2:**
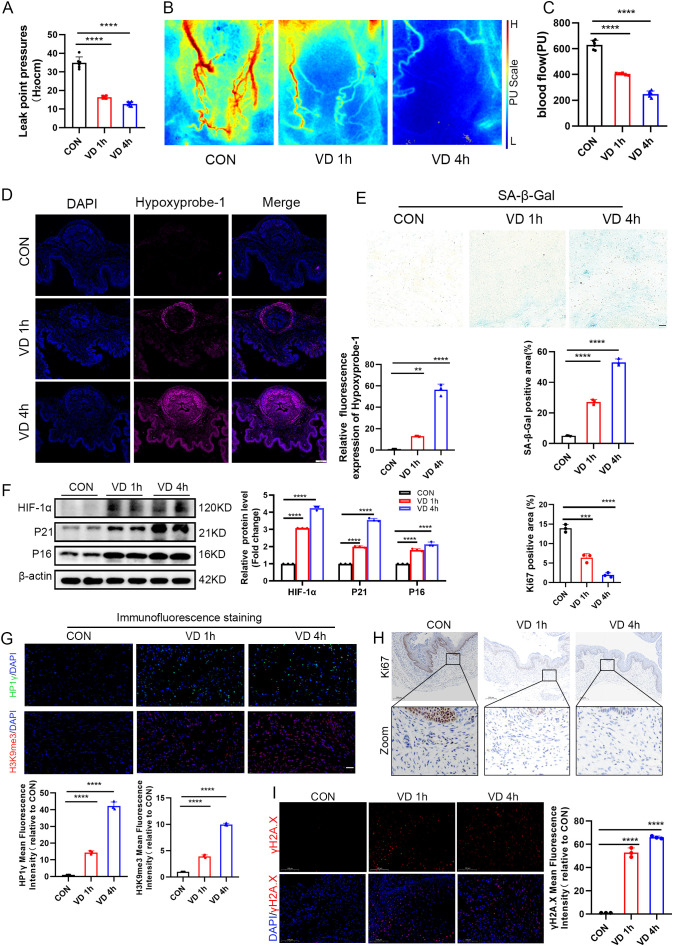
Senescence of vaginal wall cells in stress urinary incontinence model mice. (A) Quantitative analysis of bladder leak point pressure (BLPP) measurements across experimental groups (n = 6 mice per group). (B,C) Representative laser speckle contrast imaging (LSCI) of vaginal wall blood flow, accompanied by corresponding quantification (n = 6 mice per group). (D) Hypoxyprobe-1 immunostaining (red) illustrating tissue hypoxia, with quantitative assessment relative to controls (scale bar: 200 μm; n = 3 mice per group). (E) Senescence-associated β-galactosidase (SA-β-gal) staining (blue) and quantitative evaluation of senescent cells (scale bar: 20 μm; n = 3 mice per group). (F) Western blot analysis and densitometric quantification of HIF-1α, P21, and P16 protein expression, normalized to control levels (n = 3 mice per group). (G) Immunofluorescence staining was performed to visualize HP1γ (green), H3K9me3 (red), and DAPI (blue) in sections of the vaginal wall, followed by a quantitative analysis of fluorescence intensity (scale bar: 20 μm; n = 3 mice per group). (H) Ki67 immunohistochemical staining was conducted to assess proliferative activity quantitatively (scale bar: 200 μm; n = 3 mice per group). (I) γH2A.X immunofluorescence staining was utilized to quantify DNA damage (scale bar: 100 μm; n = 3 mice per group). The experimental groups included the control (CON), 1-hour vaginal distension (VD 1h), and 4-hour vaginal distension (VD 4h). Statistical significance was determined, with ***P < 0.001 and ****P < 0.0001 compared to the control group.

To systematically examine the mechanisms of injury to pelvic floor tissues during simulated childbirth, we developed a mouse model of SUI using vaginal balloon dilation (VD) and performed comprehensive evaluations. Urodynamic assessments indicated that a 1-hour VD treatment significantly reduced bladder leak point pressures (BLPP), with a further progressive decline in BLPP observed when the treatment duration was extended to 4 hours (Fig 2A). Laser speckle contrast imaging revealed a substantial decrease in vaginal wall blood perfusion following VD, which positively correlated with the duration of VD (Fig 2B, C). The detection of HypoxyprobeTM-1 and the analysis of HIF-1α protein expression revealed progressively worsening hypoxia in the anterior vaginal wall tissues with extended vaginal distension (VD) treatment (Fig 2D, F). These findings collectively suggest that simulated childbirth induces microcirculatory dysfunction and progressive hypoxic injury in the vaginal wall, implicating hypoxia as a potential factor contributing to damage in pelvic floor support tissues during VD. Given that tissue injury is a critical factor in driving cellular senescence, and that hypoxic injury is known to accelerate this process [27,28], our subsequent investigations identified localized β-galactosidase-positive cells in the stromal layer of the vaginal wall after 1 hour of VD treatment. Notably, there was a significant expansion of senescent areas following 4 hours of treatment (Fig 2E). Western blot analysis revealed that VD induced the upregulation of senescence markers P16 and P21 (Fig 2F). The formation of SAHF, which play a crucial role in cellular senescence by silencing proliferation-related genes, was confirmed through immunofluorescence detection. This detection demonstrated VD-induced accumulation of heterochromatin markers HP1γ and H3K9me3 in vaginal tissues (Fig 2G). Concurrently, a significant reduction in the expression of the proliferation marker Ki67 (Fig 2H) and an increase in the DNA damage marker γH2A.X (Fig 2I) further validated the cellular senescence phenotype. Collectively, these findings systematically demonstrate that simulated childbirth induces microcirculatory dysfunction, tissue hypoxia, and DNA damage in the vaginal wall, thereby promoting SAHF-mediated cellular senescence. This process may represent a critical pathological mechanism underlying the functional impairment of pelvic floor support tissues.

### 3.2. PIM1 is activated in both the VD mouse model and the in vitro cellular hypoxia model

**Fig 3:**
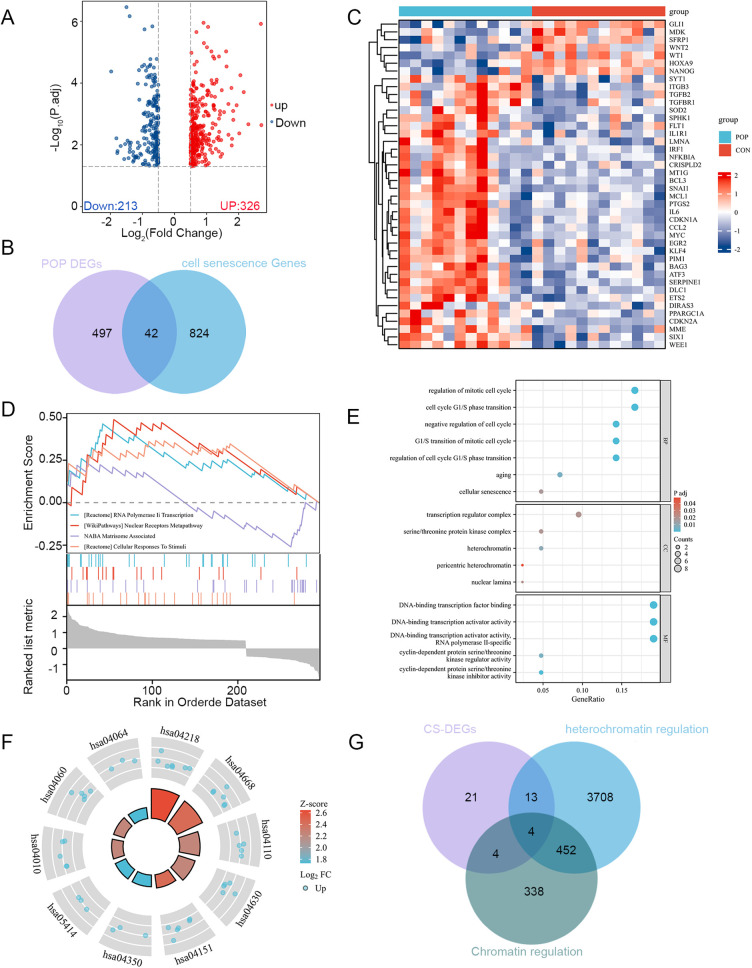
Identification of PIM1 as a key cellular senescence-related regulator differentially expressed in SUI. (A) The volcano map of GSE53868. (B) Venn diagram show that 42 overlapping cellular senescence-differentially expressed genes (CS-DEGs) in GSE53868 and CellAge database. (C) Heat map of overlapping CS-DEGs. Upregulated genes are marked in red; downregulated genes are marked in blue. (D) The top four items enriched by GSEA. (E) Enrichment result of overlapping cellular senescence‐differentially expressed genes (CS‐DEGs) Gene Ontology (GO) term. (F) Enrichment result of overlapping CS‐DEGs KEGG pathway. Adjusted p‐value <0.05 was considered significant.(G) Venn diagram show that 4 overlapping gene in CS-DEGs, heterochromatin regulation and chromatin regulation.

**Fig 4:**
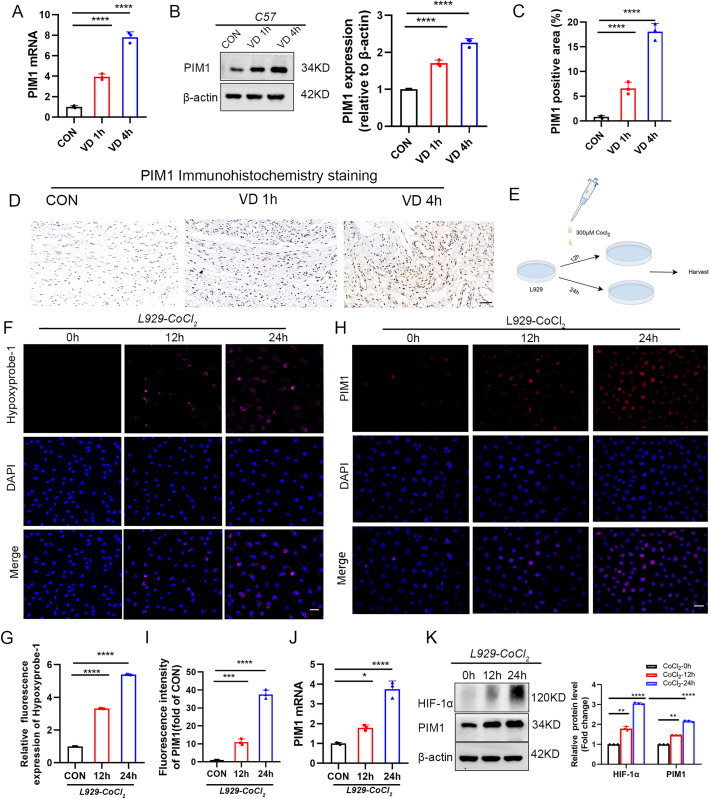
Up-regulation of PIM1 expression in in vivo and in vitro models. (A) Relative mRNA expression of PIM1 in vaginal wall tissues (n = 3). (B) Protein expression of PIM1 in vaginal wall tissues and quantitative analysis, quantitative analysis is expressed as a relative value to control (n = 3). (C,D) Representative images of immunohistochemical staining of PIM1 in vaginal wall tissues and quantitative analysis, quantitative analysis is expressed as a relative value to control (scale bar: 20μm, n = 3). e: Pattern image of cellular hypoxia model. 300μM CoCl_2_ was treated with L929 cells for 12 weeks respectively, = 3). (E) Schematic representation of the chemical hypoxia induction model in cultured cells. 300 μM CoCl_2_ treated L929 cells for 12 h and 24 h, respectively. con: control cells, 12h: CoCl_2_ treated L929 cells for 12 h group, 24h: CoCl_2_ treated L929 cells for 24 h group. (F, G) representative images and statistical analysis of hypoxia probe (Hypoxyprobe-1) staining representative images and statistical graphs. Statistical graphs are represented as relative quantification compared to control (scale bar: 20 μm, n = 3). (H,I)Representative images and statistical graphs of PIM1 immunofluorescence staining of different subgroups of cells. Statistical graphs are represented as relative quantification compared to control (scale bar: 20 μm, n = 3). (J)Relative mRNA expression of PIM1 in different subgroups of cells (n = 3). (K) Representative images and statistical graphs of immunofluorescence staining of PIM1and HIF-1α in different subgroups of cells. Statistical graphs are represented as relative quantification compared to control (n = 3). Statistical significance was determined, with **P < 0.01,***P < 0.001 and ****P < 0.0001 compared to the control group.

To investigate the mechanism of cellular senescence in the pathogenesis of SUI, we analyzed the GSE53868 dataset, which shares a similar pathogenesis with SUI. After normalizing the microarray data, we identified 539 differentially expressed genes (DEGs) in the GSE53868 dataset (Fig 3A). Through Venn diagram analysis, we identified 42 overlapping senescence-associated differentially expressed genes (CS-DEGs) between the GSE53868 dataset and 866 cellular senescence-associated genes from the CellAge database [21] (Fig 3B). Among these 42 CS-DEGs, 35 genes were up-regulated and 7 genes were down-regulated. The expression heatmap of these 42 CS-DEGs effectively distinguished the control group from the POP group (Fig 3C). Detailed information on these overlapping CS-DEGs is provided in Supplementary Table 2 (S2 Table).Gene set enrichment analysis (GSEA) revealed enrichment in RNA transcription, nuclear receptor pathway, extracellular matrix, and cellular response to stimuli (Fig 3D). To further analyze the biological functions and pathways of CS-DEGs, we conducted gene ontology (GO) and Kyoto Encyclopedia of Genes and Genomes (KEGG) pathway enrichment analyses. The GO analysis showed significant enrichment of cellular senescence and negative cell cycle regulation in the biological process (BP). In terms of cellular components (CC), DEGs were associated with heterochromatin, transcriptional regulatory complexes, and serine/threonine protein kinase complexes. Molecular function (MF) analysis indicated associations with DNA-binding transcription factor binding and cell cycle protein-dependent protein serine/threonine kinase inhibitor activity (Fig 3E and Supplementary Table 3 (S3 Table)).KEGG pathway analysis highlighted involvement in cellular senescence, TNF signaling pathway, and cell cycle (Fig 3F and Supplementary Table 3 (S3 Table)). Notably, heterochromatin-associated signals were enriched in protein-protein interactions (PPIs). By intersecting CS-DEGs with chromatin-regulated and heterochromatin-regulated genes, we identified four senescence-associated chromatin-regulated genes: PIM1, KLF4, LMNA, and SNAI1. PIM1 and KLF4 promoted cellular senescence, while LMNA and SNAI1 inhibited it; all four genes were up-regulated in the POP group (Fig 3G). Further literature review revealed that PIM1 kinase, a constitutively active serine/threonine kinase, plays a crucial role in cell cycle regulation, cell survival, anti-apoptosis, and energy metabolism, with implications in cellular senescence [17]. To validate our findings, experiments were conducted using the SUI mouse model. The RT-qPCR analysis revealed a significant upregulation in PIM1 mRNA expression in the vaginal wall of mice treated with VD (Fig 4A). Consistently, Western Blot and immunohistochemistry results demonstrated higher PIM1 expression levels in the VD group compared to the CON group, with a gradient increase correlating with the duration of VD (Fig 4B–D).

**Fig 5:**
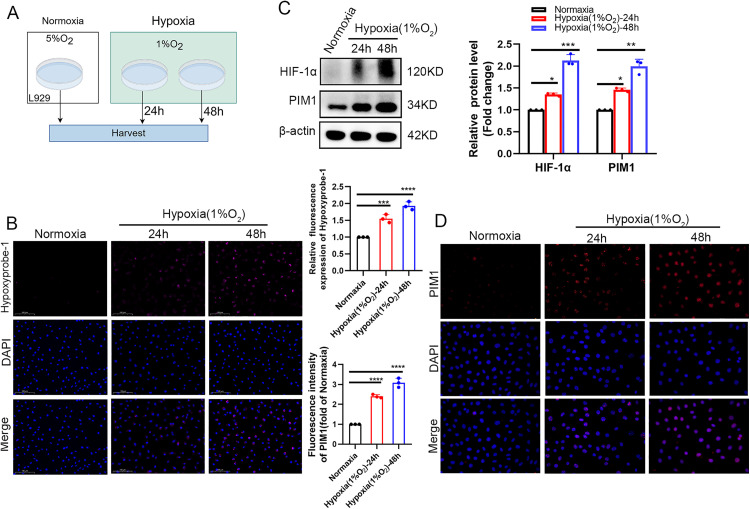
Upregulation of PIM1 expression in hypoxia chamber models. (A) Schematic diagram of the hypoxia chamber system for establishing cellular hypoxia (1% O₂) in L929 cells. Experimental groups: normoxic control (21% O₂), 24-h hypoxia, and 48-h hypoxia. (B) Representative immunofluorescence images and quantitative analysis of Hypoxyprobe-1 staining (red) demonstrating progressive hypoxia development (scale bar: 20 μm; n = 3 independent experiments). Data normalized to normoxic control. (C) Western blot analysis with densitometric quantification showing time-dependent upregulation of PIM1 and HIF-1α protein expression (n = 3 biological replicates). (D) Immunofluorescence staining and quantitative assessment of PIM1 expression (green) under different oxygen conditions (scale bar: 20 μm; n = 3 technical replicates). Statistical significance was determined, with ****P < 0.0001 compared to the control group.

To further investigate the regulatory mechanisms of PIM1 under hypoxic conditions, we first established an in vitro fibroblast hypoxia model by treating L929 cells with 200 μM CoCl₂ (Fig 4E). Hypoxyprobe-1 detection and HIF-1α protein analysis confirmed progressively intensified intracellular hypoxia with prolonged CoCl₂ treatment (Fig 4F, G, K), validating successful model establishment. RT-qPCR, Western blot, and immunofluorescence analyses demonstrated time-dependent upregulation of PIM1 expression at both mRNA and protein levels in CoCl₂-treated fibroblasts compared to normoxic controls (Fig 4H–K), suggesting that the observed PIM1 elevation in vaginal wall tissues of SUI mice might be driven by hypoxic microenvironment. To better mimic physiological hypoxia, we employed a 1% O₂ hypoxia workstation with three experimental groups: normoxia control, 24-hour hypoxia, and 48-hour hypoxia (Fig 5A). Both Hypoxyprobe-1 assay and HIF-1α expression analysis confirmed stable hypoxia induction (Fig 5B, C). Western blot revealed significant hypoxia duration-dependent increase in PIM1 protein expression (Fig 5C), while immunofluorescence analysis showed consistent subcellular localization patterns of PIM1 between chemical and physical hypoxia models, with hypoxia significantly enhancing PIM1 fluorescence intensity (Fig 5D). Collectively, these complementary models demonstrate that hypoxic microenvironment dynamically regulates PIM1 expression in fibroblasts, implicating its potential role as a key effector molecule in hypoxia stress response during SUI pathogenesis.

### 3.3. PIM1 regulates fibroblast SAHF formation and promotes cellular senescence

**Fig 6:**
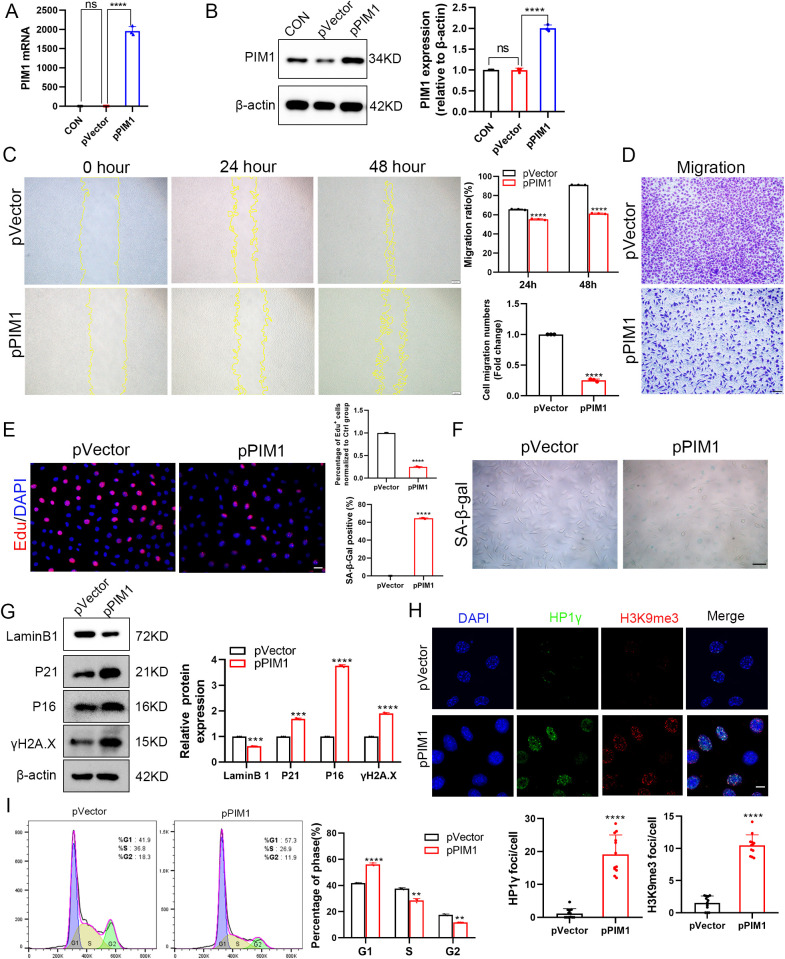
PIM1 overexpression promotes senescence in L929 cells. (A)PIM1 mRNA expression in L929 cells transfected with the PIM1 overexpression plasmid was detected using RT-qPCR. (B)Expression and quantitative analysis of PIM1 protein in L929 cells transfected with the PIM1 overexpression plasmid were determined by Western blot. (C) Representative images (left) and quantitative analysis (right) of wound healing assays in cells with PIM1 overexpression (scale bar: 100μm). (D) Transwell migration assay showing representative images (right) and quantification (left) of migrated cells(scale bar: 100μm). (E)Proliferation capacity of L929 cells transfected with the PIM1 overexpression plasmid was assessed using EDU staining and quantitatively analyzed (scale bar: 20μm). (F)SA-β-gal staining and quantitative analysis of L929 cells transfected with the PIM1 overexpression plasmid were performed (scale bar: 50μm). (G)Expression and quantitative analysis of LaminB1, P21, P16, γH2A.X, and β-actin proteins in L929 cells transfected with the PIM1 overexpression plasmid were determined by Western blot. (H)Laser confocal immunofluorescence images of HP1γ (green), H3K9me3 (red), and DAPI (blue) in L929 cells transfected with the PIM1 overexpression plasmid and quantification using ImageJ software (scale bar: 10μm). (I) Cell cycle analysis by flow cytometry in L929 cells transfected with PIM1 overexpression plasmid. Representative DNA histograms show cell distribution in G1, S, and G2 phases (analyzed by FlowJo software using Watson model for curve fitting).All results represent data from three independent experiments. Control group: CON, empty vector group: pVector, PIM1-expressing plasmid group: pPIM1. **P < 0.01, ***P < 0.001, ****P < 0.0001, ns: non-significant.

To elucidate the mechanistic role of PIM1 in fibroblast senescence, we first successfully constructed PIM1 overexpression plasmids and validated their expression efficiency (Fig 6A, B). EdU proliferation assays demonstrated that PIM1 overexpression significantly inhibited fibroblast proliferative capacity (Fig 6E). The wound healing assay showed comparable initial scratch widths at 0 hours across all groups. However, PIM1 overexpression significantly inhibited wound closure after 24 and 48 hours (Fig 6C). Furthermore, the transwell assay demonstrated that PIM1 overexpression markedly reduced the number of migrating cells (Fig 6D). Senescence-associated β-galactosidase staining showed significantly increased positive cell numbers following PIM1 overexpression (Fig 6F). Molecular analyses by Western blot confirmed that PIM1 overexpression concurrently upregulated cell cycle regulators P16 and P21, promoted accumulation of DNA damage marker γH2A.X, and substantially decreased nuclear envelope protein Lamin B1 expression (Fig 6G). Immunofluorescence microscopy further revealed that PIM1 overexpression not only enhanced expression of heterochromatin markers H3K9me3 and HP1γ but also induced their characteristic nuclear aggregation (Fig 6H), indicative of SAHF formation. Additionally, flow cytometry analysis demonstrated PIM1-mediated cell cycle arrest at G1 phase, evidenced by increased G1 population with corresponding decreases in S and G2 phases (Fig 6I). These comprehensive experimental findings collectively establish that PIM1 orchestrates multiple senescence-promoting mechanisms – including cell cycle arrest, DNA damage response, nuclear lamina disruption, and heterochromatin reorganization – to effectively drive fibroblast senescence progression.

### 3.4. Downregulation of PIM1 expression reduces SAHF formation and alleviates hypoxia-induced fibroblast senescence

**Fig 7:**
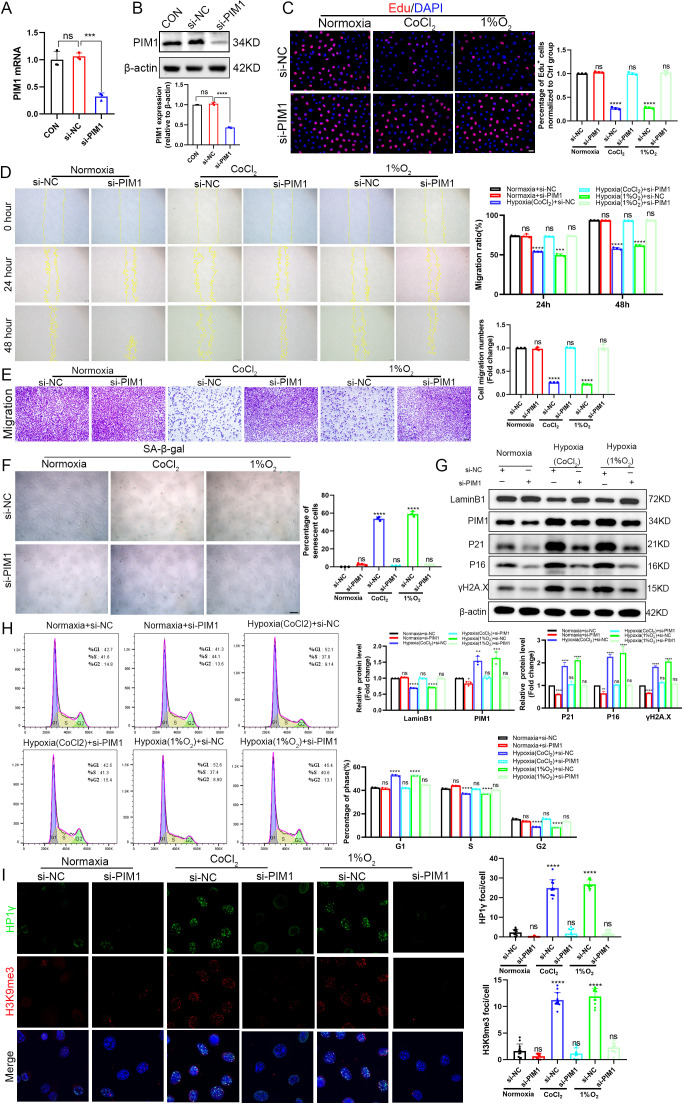
Genetic inhibition of PIM1 rescues hypoxia-induced fibroblast senescence. (A-B) Knockdown efficiency of PIM1 siRNA (si-PIM1) in L929 cells assessed by RT-qPCR (A) and Western blot (B) at 24h post-transfection, compared with negative control siRNA (si-NC). (C) EdU incorporation assay evaluating proliferative capacity of L929 cells following PIM1 knockdown and subsequent treatment with 300μM CoCl₂ for 24h or 1% O₂ for 48h (scale bar: 20μm). (D) Wound healing assay measuring migratory ability under different treatment conditions (scale bar: 100μm).(E) Transwell migration assay showing representative images (left) and quantification (right) of migrated cells(scale bar: 100μm). (F) SA-β-gal staining and quantification of senescent cells across experimental groups (scale bar: 50μm). (G) Western blot analysis of senescence-associated markers (Lamin B1, PIM1, P21, P16, γH2A.X) with β-actin loading control, including densitometric quantification. (H) Flow cytometric analysis of cell cycle distribution (G1, S, G2 phases) using Watson model-based curve fitting in FlowJo software. (I) Confocal immunofluorescence images showing HP1γ (green), H3K9me3 (red), and DAPI (blue) localization in PIM1-knockdown cells (scale bar: 10μm), with quantitative analysis by ImageJ. All results represent data from three independent experiments. **P < 0.01, ***P < 0.001, ****P < 0.0001, ns: non-significant.

To establish the pivotal role of PIM1 in hypoxia-induced fibroblast senescence, we effectively knocked down PIM1 expression via siRNA transfection prior to either CoCl₂ treatment or 1% O₂ hypoxia chamber exposure (Fig 7A, B). Functional assays demonstrated that hypoxia treatment significantly suppressed fibroblast proliferative activity, while PIM1 knockdown markedly reversed this proliferation inhibition (Fig 7C). The wound healing assay revealed that hypoxic conditions significantly impaired cell migration capacity, as evidenced by a markedly delayed wound closure rate. In contrast, PIM1 knockdown substantially ameliorated the hypoxia-induced migration defect and promoted wound closure (Fig 7D). This finding was further validated by the Transwell assay, which demonstrated that hypoxia significantly suppressed the transmembrane migration ability of cells compared with the normoxic control, whereas PIM1 knockdown effectively reversed this inhibitory effect (Fig 7E). Senescence-associated β-galactosidase staining revealed that PIM1 knockdown effectively attenuated the hypoxia-induced increase in senescence-positive cells (Fig 7F). Mechanistic investigations showed that hypoxia-mediated upregulation of P16, P21 and γH2A.X expression along with Lamin B1 downregulation were all reversed by PIM1 knockdown (Fig 7G). Cell cycle analysis verified that PIM1 silencing alleviated hypoxia-induced G1 phase arrest (Fig 7H). Immunofluorescence detection of H3K9me3 and HP1γ demonstrated that hypoxia promoted formation of characteristic SAHF heterochromatin foci, whereas PIM1 knockdown significantly reduced the aggregation of these senescence-associated chromatin structures (Fig 7I). These comprehensive results conclusively demonstrate that PIM1 plays a central role in hypoxia-induced fibroblast senescence by regulating SAHF formation, and that targeted inhibition of PIM1 effectively mitigates hypoxia-associated cellular senescence phenotypes.

### 3.5. The PIM1 inhibitor AZD-1208 attenuates SAHF formation and ameliorates hypoxia-induced fibroblast senescence

**Fig 8:**
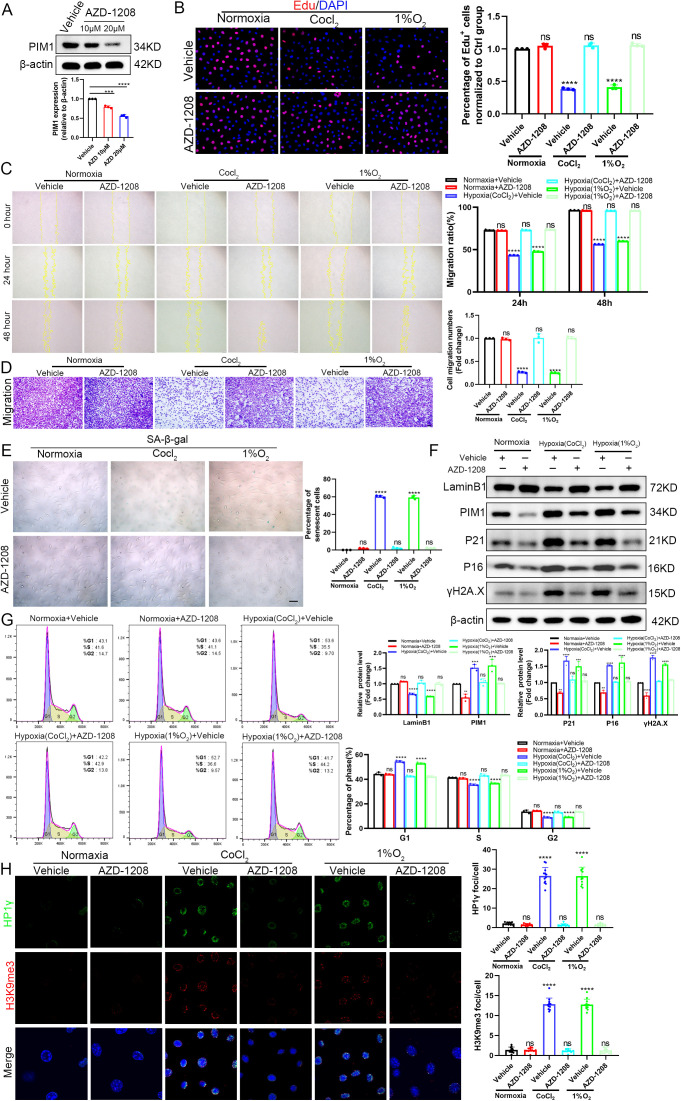
Pharmacological inhibition of PIM1 by AZD-1208 ameliorates hypoxia-induced fibroblast senescence. (A) Dose-dependent inhibition of PIM1 by AZD-1208 (10μM and 20μM for 24h) assessed by Western blot, with vehicle-treated cells as control. The 20μM concentration was selected for subsequent experiments. (B) EdU incorporation assay demonstrating proliferative capacity of L929 cells following AZD-1208 treatment combined with either 300μM CoCl₂ (24h) or 1% O₂ (48h) exposure (scale bar: 20μm). (C) Wound healing assay quantifying cell migration under different treatment conditions (scale bar: 200μm). (D) Transwell migration assay showing representative images (left) and quantification (right) of migrated cells(scale bar: 100μm). (E) SA-β-gal staining and quantitative analysis of cellular senescence (scale bar: 50μm). (F) Western blot analysis of senescence-associated markers (Lamin B1, PIM1, P21, P16, γH2A.X) with β-actin loading control, including densitometric quantification. (G) Flow cytometric cell cycle profiling (G1, S, G2 phases) analyzed using FlowJo software with Watson model-based curve fitting. (H) Confocal microscopy images showing subcellular localization of HP1γ (green), H3K9me3 (red), and DAPI (blue) in AZD-1208-treated cells (scale bar: 10μm), with ImageJ-based quantification. All results represent data from three independent experiments. **P < 0.01, ***P < 0.001, ****P < 0.0001, ns: non-significant.

To investigate the therapeutic potential of PIM1 inhibition in hypoxia-induced fibroblast senescence, we employed the specific PIM1 inhibitor AZD-1208 in both chemical (CoCl₂) and physical (1% O₂) hypoxia models (Fig 8A). Experimental results demonstrated that AZD-1208 treatment significantly alleviated hypoxia-induced suppression of fibroblast proliferation (Fig 8B). The wound healing assay indicated that hypoxia significantly suppressed cell migratory activity, whereas treatment with AZD-1208 markedly reversed this inhibitory phenotype, as reflected by a significantly accelerated wound closure rate (Fig 8C). The Transwell assay further confirmed this conclusion, demonstrating that AZD-1208 effectively restored the hypoxia-impaired transmembrane migration capacity of cells (Fig 8D). Senescence-associated β-galactosidase staining confirmed that AZD-1208 effectively reduced the hypoxia-induced increase in senescence-positive cells (Fig 8E). At the molecular level, Western blot analysis showed AZD-1208 treatment reversed hypoxia-mediated upregulation of P16, P21 and γH2A.X expression while restoring Lamin B1 levels (Fig 8F). Flow cytometry analysis verified that AZD-1208 attenuated hypoxia-induced G1 phase cell cycle arrest (Fig 8G). Furthermore, immunofluorescence detection demonstrated AZD-1208 significantly reduced hypoxia-promoted nuclear aggregation of H3K9me3 and HP1γ, indicating its inhibitory effect on SAHF formation (Fig 8H). These findings not only further validate the crucial role of PIM1 in hypoxia-induced cellular senescence but, more importantly, demonstrate that the specific PIM1 inhibitor AZD-1208 exhibits therapeutic potential for ameliorating hypoxia-associated cellular senescence through modulation of multiple senescence-related pathways.

### 3.6. AZD-1208 ameliorates stress urinary incontinence by targeting PIM1 to attenuate cellular senescence

**Fig 9:**
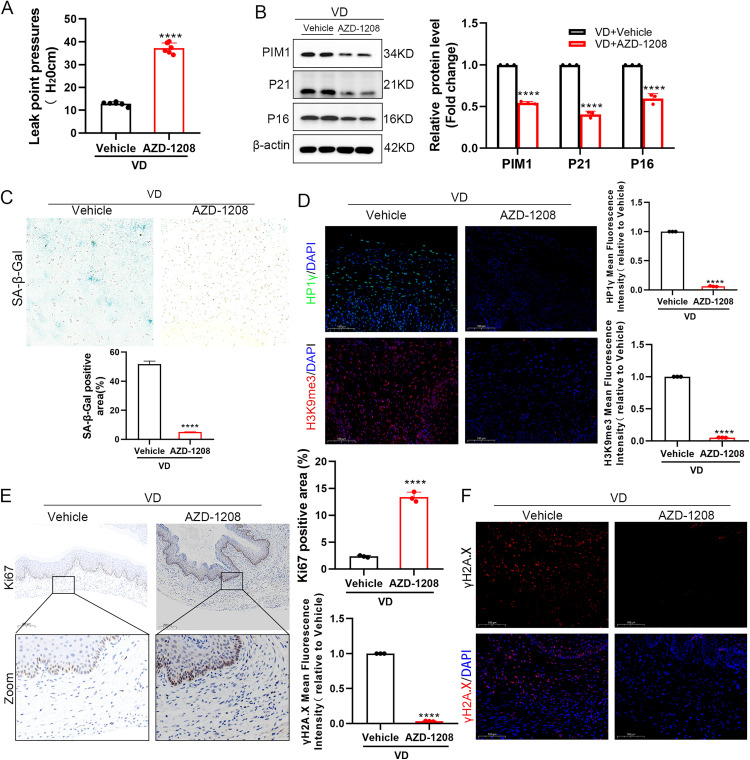
Therapeutic efficacy of AZD-1208 in alleviating cellular senescence and improving stress urinary incontinence. (A) AZD-1208 pretreatment (40 mg/kg/day, i.p.) initiated 3 days prior to vaginal distension (VD) and continued throughout the study period. Quantitative analysis of bladder leak point pressure (BLPP) demonstrates functional recovery. (B) Western blot analysis with densitometric quantification of HIF-1α, P21, and P16 protein expression in vaginal tissues normalized to β-actin (n = 3 mice/group). (C) SA-β-gal staining (blue) and quantitative assessment of senescent cell burden (scale bar: 20 μm; n = 3). (D) Immunofluorescence staining of HP1γ (green), H3K9me3 (red), and DAPI (blue) in vaginal wall sections with quantitative intensity analysis (scale bar: 20 μm; n = 3). (E) Ki67 immunohistochemistry assessing proliferative capacity (scale bar: 200 μm; n = 3). (F) γH2A.X immunofluorescence revealing DNA damage repair (scale bar: 100 μm; n = 3). All results represent data from three independent experiments. **P < 0.01, ***P < 0.001, ****P < 0.0001.

To elucidate the translational value of PIM1 inhibition in SUI treatment, we conducted interventional studies using the specific PIM1 inhibitor AZD-1208. A systematic therapeutic evaluation system was established by initiating intraperitoneal AZD-1208 pretreatment three days prior to vaginal distension (VD) modeling. Urodynamic analyses demonstrated that AZD-1208 pretreatment significantly ameliorated the VD-induced reduction in bladder leak point pressure (BLPP) (Fig 9A), indicating its therapeutic potential for restoring pelvic floor function. At the molecular level, Western blot confirmed AZD-1208 effectively normalized VD-induced aberrant expression of senescence markers P16 and P21 in vaginal wall tissues (Fig 9B). Histopathological analysis revealed a marked decrease in β-galactosidase-positive cells in AZD-1208-treated groups (Fig 9C), with immunofluorescence demonstrating significant inhibition of heterochromatin aggregation (HP1γ and H3K9me3) (Fig 9D), suggesting intervention in SAHF formation. Notably, AZD-1208 not only enhanced expression of proliferation marker Ki67 (Fig 9E) but also reduced levels of DNA damage marker γH2A.X (Fig 9F). These findings systematically reveal that AZD-1208 plays a crucial role in SUI treatment through coordinated modulation of multiple mechanisms including cellular senescence, proliferative activation, and DNA damage repair.

## 4. Discussion

SUI constitutes a significant global health concern affecting women, with its rising prevalence garnering considerable attention. Although surgical interventions, such as midurethral slings, are the primary treatment modality, their therapeutic efficacy exhibits substantial interindividual variability and may be associated with complications, including bladder perforation and urinary retention [29]. Therefore, elucidating the pathophysiological mechanisms underlying SUI is of considerable clinical importance for the development of novel targeted therapies. Current evidence suggests that vaginal delivery, a major risk factor for SUI, induces hypoxic damage to pelvic floor supportive tissues, which plays a crucial role in the pathogenesis of the condition. Importantly, while the association between tissue hypoxia and cellular senescence has been established in various disease models, a systematic investigation of this mechanism in the context of SUI is still lacking.

Epidemiological research has established a definitive link between vaginal delivery and the onset of SUI. A multicenter cohort study indicated that approximately 51.5% of pregnant women experienced SUI symptoms during pregnancy, and 12.5% of those who underwent vaginal delivery continued to exhibit persistent urinary incontinence at 12 months postpartum. This prevalence is notably higher than the 7.2% observed in women who delivered via cesarean section [30]. Furthermore, increased parity has been recognized as a contributing factor to the development of SUI. Women with multiple vaginal deliveries demonstrate a higher incidence of urinary incontinence compared to those who delivered via cesarean section.The cumulative impact of repeated vaginal deliveries exacerbates pelvic floor dysfunction, resulting in a heightened prevalence of SUI among multiparous women [31]. Moreover, factors such as macrosomia, prolonged second stage of labor, and multiple vaginal deliveries have been associated with both pelvic floor injury and the occurrence of SUI [9,32]. The concurrence of these clinical events suggests a shared mechanism of pelvic organ and tissue injury, potentially involving ischemic and/or reperfusion damage to the pelvic floor. When intrapartum pressure surpasses the ischemic threshold of peripheral nerves (80 mmHg)—with peak pressures during vaginal delivery reaching up to 200 mmHg [33]—it leads to microcirculatory disturbances within the pelvic floor tissues. In our study, utilizing laser speckle contrast imaging (LSCI) technology, we demonstrated for the first time in a vaginal distension (VD) mouse model that: 1) a 1-hour VD was sufficient to cause a significant reduction in vaginal wall blood perfusion, and 2) extending the duration to 4 hours further intensified hypoxic injury. These findings align with previous reports by Liang et al. [34] which documented decreased urethral microvascular density in patients with SUI, collectively underscoring the critical role of hypoxic injury in the pathophysiology of SUI. Research has demonstrated that hypoxic injury leads to a range of structural and functional changes in urethral supportive tissues through various mechanisms, including oxidative stress, extracellular matrix remodeling, inflammatory responses, and the loss of muscular integrity [35–37] These alterations are crucial for maintaining continence and overall bladder function. Recent studies have reported a significantly elevated expression of hypoxia-inducible factor 1-alpha (HIF-1α) in the vaginal wall tissues of patients with pelvic organ prolapse (POP), indicating that hypoxic injury may play a significant role in the pathogenesis of POP [38]. Our study similarly observed a progressive increase in HIF-1α expression in the vaginal wall tissues of SUI mice subjected to prolonged vaginal distension (VD). Investigations using animal models predisposed to POP have revealed a failure of cellular mechanisms that typically prevent senescence following vaginal distension. In line with these findings, we observed characteristics of cellular senescence in VD model mice, evidenced by increased β-galactosidase activity and the formation of SAHF. Collectively, these results suggest that hypoxia-induced cellular senescence may represent a critical factor in the pathophysiology of these conditions.

To further elucidate the mechanisms underlying cellular senescence in the pathogenesis of SUI, we concentrated on PIM1, a serine/threonine protein kinase known to regulate a variety of cellular functions. Recent evidence has increasingly highlighted the role of PIM1 in facilitating cellular senescence [18,39]. In prostate cancer cell lines, PIM1 has been demonstrated to induce senescence, as indicated by increased β-galactosidase activity (SA-β-Gal) and elevated P21 expression [40].More recently, Yang et al. [41] eported that PIM1 destabilizes UHRF1, resulting in DNA hypomethylation, genomic instability, increased P16 expression, and subsequent cellular senescence [41]. At the molecular level, our findings elucidate hypoxia-induced cellular senescence as a pivotal intermediary between mechanical injury and pelvic floor dysfunction. We observed that vaginal distension (VD) not only upregulated HIF-1α expression in vaginal wall tissues but also enhanced β-galactosidase activity and SAHF formation. These results are consistent with prior reports of increased HIF-1α expression in vaginal tissues from patients with POP [38] indicating that hypoxia-associated senescence may constitute a common pathological mechanism underlying pelvic floor disorders. Importantly, we have identified for the first time that PIM1 kinase serves as a central regulatory element in this process. Consistent with established literature, our data confirm that P21 expression is associated with senescence across multiple cell types, particularly in response to DNA damage or other cellular stressors [42].Furthermore, the accumulation of γH2A.X, a well-characterized marker of DNA damage, reflects persistent genomic instability in senescent cells [43].Our results demonstrate that under hypoxic conditions, PIM1 expression increases in a time-dependent manner and promotes cellular senescence through dual mechanisms: 1) upregulating cell cycle inhibitors such as P16 and P21, and 2) impairing DNA damage repair via SAHF formation. This “two-pronged” mechanism explains why PIM1 overexpression so effectively induces a senescent phenotype.

It is generally accepted that chromatin surrounding DNA damage sites needs to maintain an “open” configuration to facilitate the recruitment of repair factors and subsequent damage repair [44].Consequently, the formation of heterochromatin during senescence likely creates a physical barrier that prevents DNA repair machinery from accessing damaged sites, thereby perpetuating the DNA damage response (DDR). Our findings demonstrate that PIM1 upregulation promotes SAHF formation, which in turn sustains DDR activation. SAHF are known to mediate irreversible cell cycle arrest in senescent cells by repressing proliferation-promoting genes such as CCNA2 [12]. During cellular senescence, cells undergo profound molecular alterations that not only compromise their proliferative capacity but also exert detrimental effects on neighboring cells and tissues through paracrine signaling [45]. Notably, the accumulation of senescent cells has been closely associated with various age-related pathologies, primarily mediated through the secretion of pro-inflammatory factors that disrupt tissue homeostasis [46].The molecular circuitry governing senescence involves complex crosstalk between multiple signaling pathways, with the P53/P21 and p16INK4A/pRB axes playing pivotal roles [12]. Pathway activation not only induces cell cycle arrest but also drives characteristic senescent phenotypes including SAHF formation [12]. In our experimental models, vaginal distension (VD) significantly enhanced SAHF formation in mouse vaginal walls. Similarly, prolonged hypoxia time-dependently increased SAHF accumulation in cultured fibroblasts. Importantly, both genetic (siPIM1) and pharmacological (AZD-1208) inhibition of PIM1 effectively reversed hypoxia-induced senescent phenotypes. From a translational perspective, our study highlights the therapeutic potential of PIM1 inhibition for SUI management. In vivo experiments showed that AZD-1208 pretreatment not only restored bladder leak point pressure but also markedly reduced senescent areas in vaginal tissues. At the molecular level, AZD-1208 treatment simultaneously downregulated P16/P21 expression, enhanced Ki67-positive cell proliferation, and attenuated DNA damage markers. These findings provide preclinical evidence for developing PIM1-targeted therapies for SUI. Given the established safety profile of AZD-1208 in oncology clinical trials, its repurposing for SUI treatment appears particularly promising.

The recently proposed “core senescence network” concept [47] provides a broader theoretical framework for our findings. This paradigm emphasizes the spatiotemporal coordination of multiple pathways – including P16/P21, NF-κB, and senescence-associated secretory phenotype (SASP) – during cellular senescence. Our experimental results extend this understanding by demonstrating that in pelvic floor tissues, PIM1 may serve as an upstream regulatory node that concurrently modulates chromatin remodeling (via SAHF formation) and cell cycle arrest (through P16/P21 activation) to drive senescence progression. These observations align with the established “epigenetic-metabolic reprogramming axis” [48], which posits that DNA damage-induced heterochromatinization irreversibly locks cells into a senescent state – a mechanism strongly supported by our SAHF quantification in SUI models. Emerging evidence suggests circadian rhythm disruption may accelerate cellular senescence by impairing DNA repair efficiency [49]. Although our study did not directly examine clock-related genes, clinical observations linking postpartum nocturia with SUI incidence provide rationale for exploring chronotherapeutic strategies in future research [50].

Several limitations merit consideration in this study. Firstly, although the VD mouse model effectively simulates mechanical birth trauma, it does not fully capture the intricate pathophysiology of human SUI, including aspects such as hormonal fluctuations and neural regeneration. Secondly, the pathogenesis of SUI involves multifactorial mechanisms beyond hypoxia-induced senescence, necessitating further exploration of how our findings interact with established factors such as neural injury, extracellular matrix remodeling, and estrogen deficiency. Lastly, the specific downstream effectors of PIM1-mediated senescence have yet to be elucidated. Future research utilizing single-cell sequencing technologies [51] could offer spatiotemporal insights into the role of PIM1 in pelvic floor cellular senescence.

## 5. Conclusions

In this study, we developed a SUI mouse model by simulating vaginal delivery and demonstrated the presence of hypoxic damage and cellular senescence in the vaginal wall of SUI mice. Previous research has indicated that under conditions of hypoxic injury, PIM1 facilitates fibroblast senescence by promoting the formation of SAHF. The accumulation of these senescent fibroblasts within the vaginal wall compromises the supporting tissue of the pelvic floor, ultimately resulting in the onset of SUI. Our study offers new insights into the pathogenesis of SUI through the lens of cellular senescence and aids in the development of novel therapies aimed at treating or preventing SUI, particularly following vaginal delivery. These findings carry significant scientific and clinical implications for enhancing the prevention and treatment strategies for SUI.

## Supporting information

S1 Table Primer sequences used in this study. (XLSX)

S2 Table The details of the overlapping CS-DEGs. (XLSX)

S3 Table GO and KEGG enrichment analysis of overlapping CS‐DEGs. (XLSX)

S1 File Raw images. Original uncropped and unadjusted Western blot images.
(PDF)
