# Supplementary figures and images for: PIM1 induces hypoxia-related fibroblast senescence in a mouse model of stress urinary incontinence
Source: PLoS One. 2025 Nov 12;20(11):e0335501. doi: 10.1371/journal.pone.0335501 (PMC12611163; doi:10.1371/journal.pone.0335501)

# S1\_raw\_images.

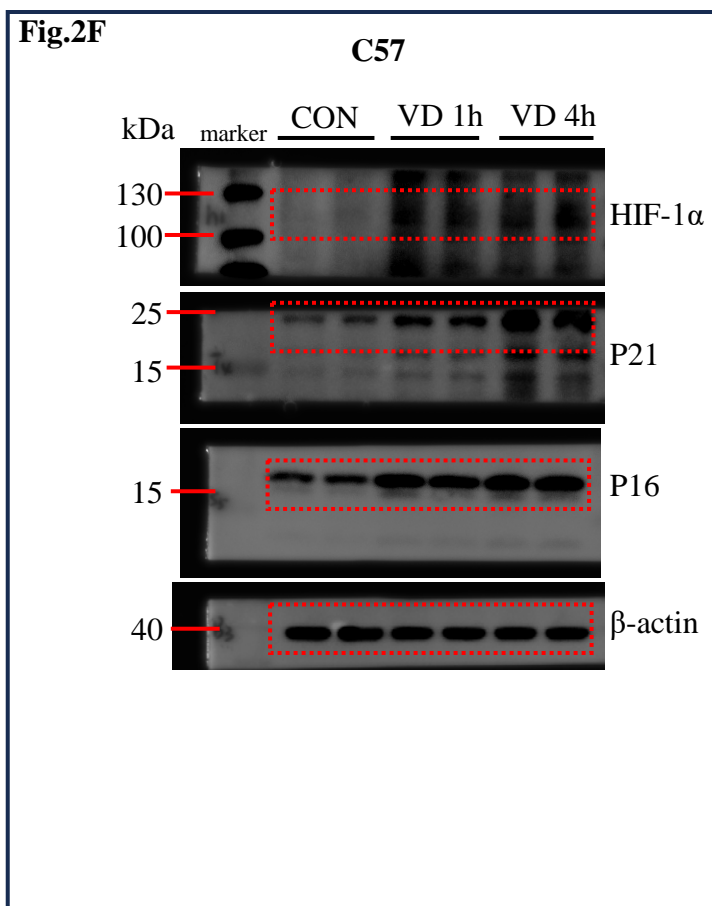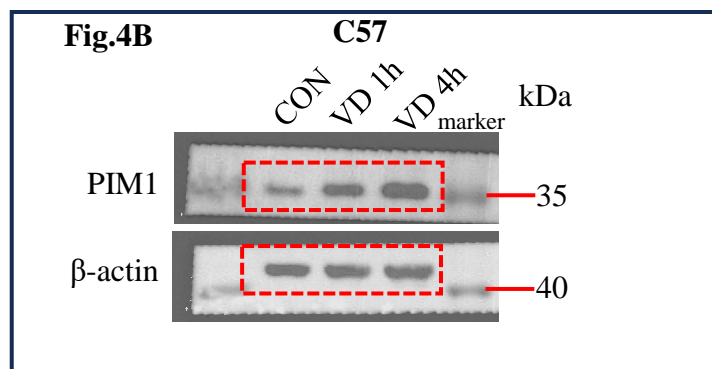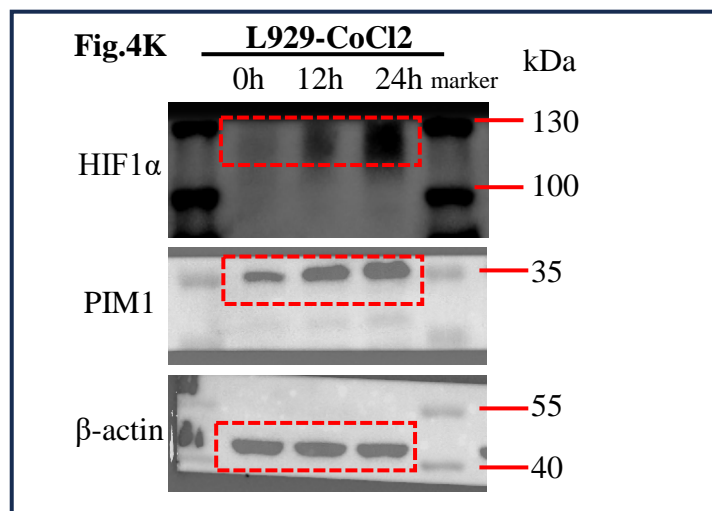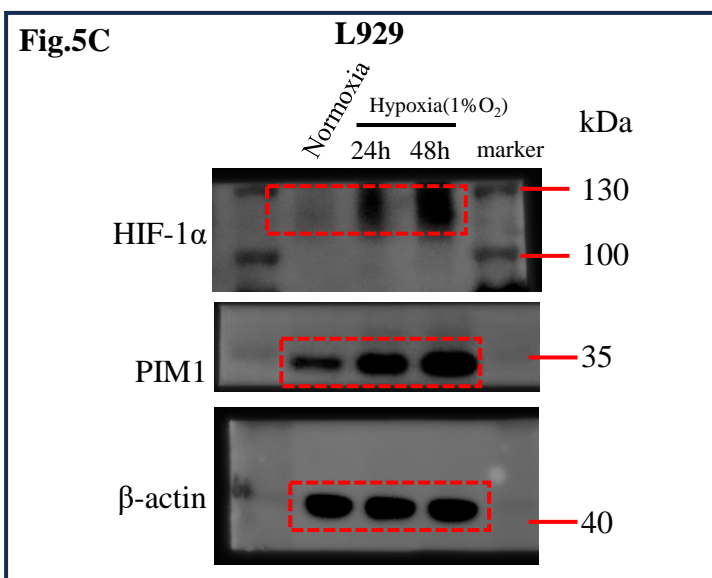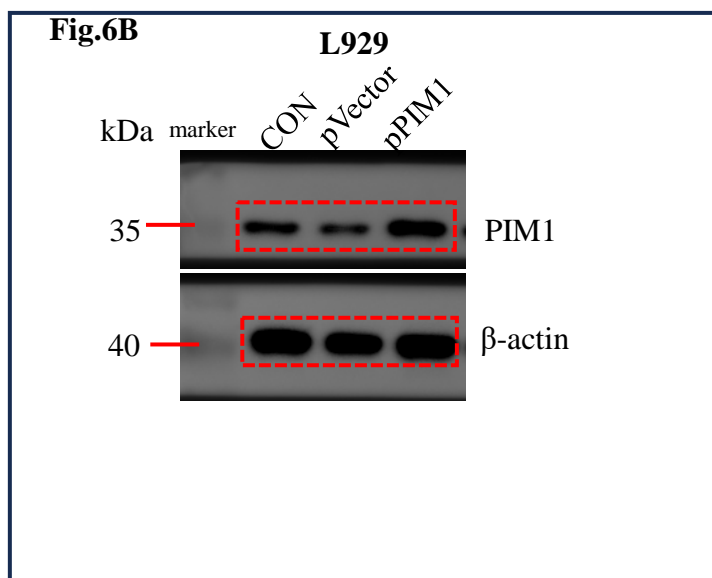

**Fig.6G**

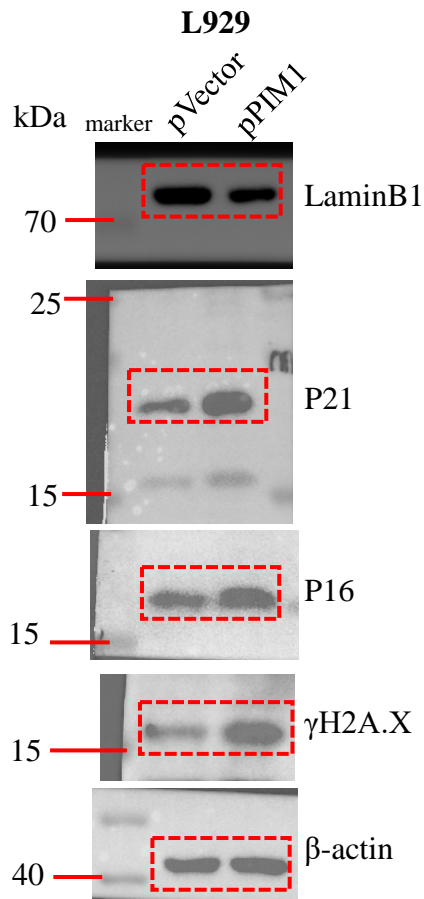

**Fig.7B**

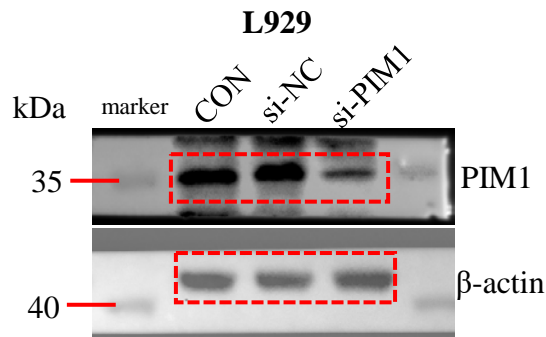

**Fig.7G**

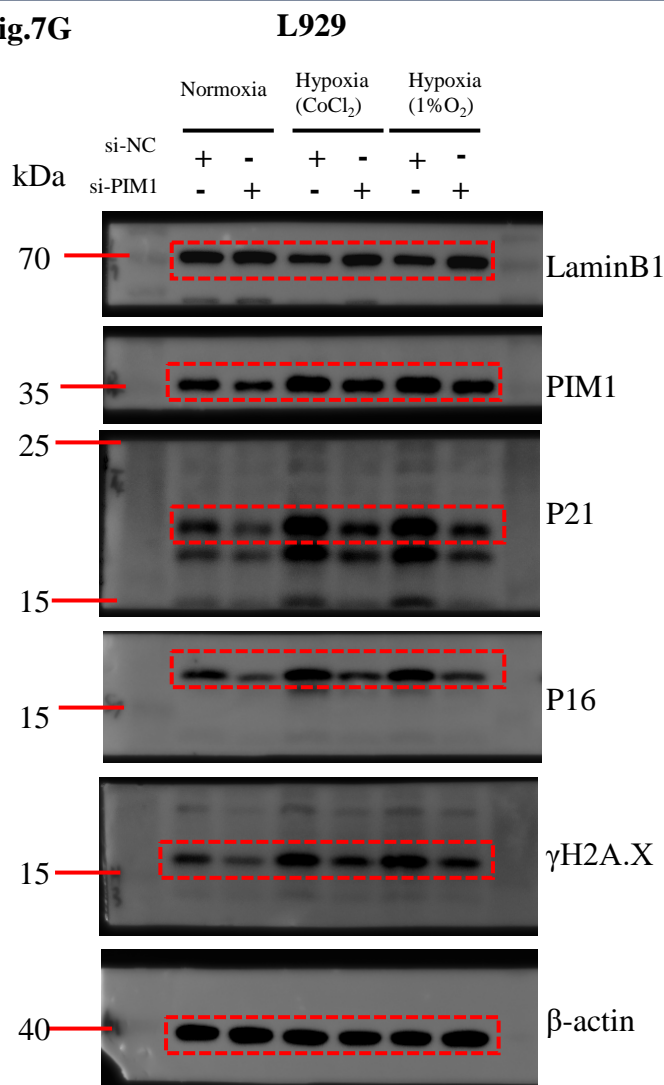

**Fig.8A**

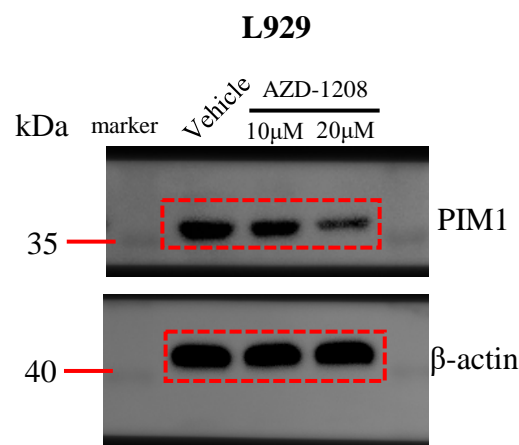

**Fig.8F** **L929**

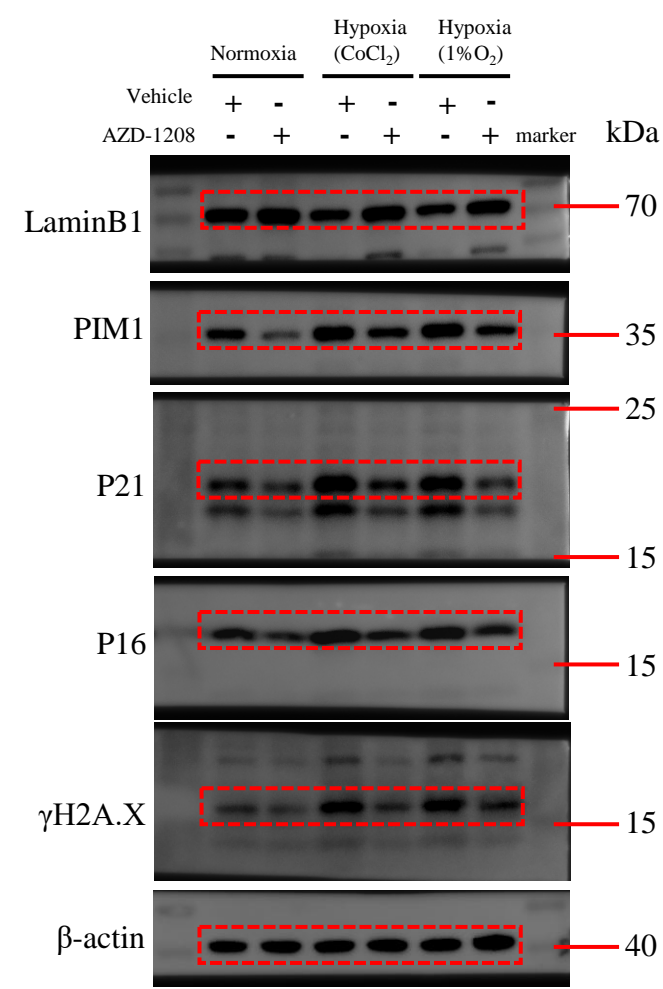

**Fig.9B**

**C57**

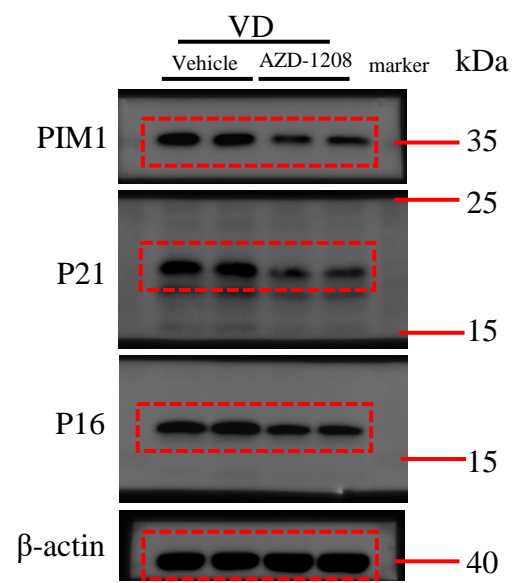

Supplement: S1 File — Original uncropped and unadjusted Western blot images. (PDF) [file pone.0335501.s004.pdf]
